# Optimal allocation of scarce PCR tests during the COVID-19 pandemic

**DOI:** 10.1371/journal.pone.0285083

**Published:** 2023-06-05

**Authors:** Afschin Gandjour

**Affiliations:** Frankfurt School of Finance & Management, Frankfurt, Germany; University of Siena: Universita degli Studi di Siena, ITALY

## Abstract

**Background/aim:**

During the coronavirus disease (COVID-19) pandemic, Germany and various other countries experienced a shortage of polymerase chain reaction (PCR) laboratory tests due to the highly transmissible SARS-CoV-2 Omicron variant that drove an unprecedented surge of infections. This study developed a mathematical model that optimizes diagnostic capacity with lab-based PCR testing.

**Methods:**

A mathematical model was constructed to determine the value of PCR testing in relation to the pre-test probability of COVID-19. Furthermore, the model derives the lower and upper bounds for the threshold pre-test probability of the designated priority group. The model was applied in a German setting using the PCR test-positivity rate at the beginning of February 2022.

**Results:**

The value function of PCR testing is bell-shaped with respect to the pre-test probability, reaching a maximum at a pre-test probability of 0.5. Based on a PCR test-positivity rate of 0.3 and assuming that at least two thirds of the tested population have a pre-test probability below, lower and higher pre-test probability thresholds are ≥ 0.1 and 0.7, respectively. Therefore, individuals who have a 25% likelihood of testing positive because they exhibit symptoms should be a higher priority for PCR testing. Furthermore, a positive rapid antigen test in asymptomatic individuals with no known exposure to COVID-19 should be confirmed using PCR. Yet, symptomatic individuals with a positive RAT should be excluded from PCR testing.

**Conclusion:**

A mathematical model that allows for the optimal allocation of scarce PCR tests during the COVID-19 pandemic was developed.

## Introduction

During the surge in COVID-19 cases due to the highly transmissible Omicron variant, many countries were facing a shortage of rapid antigen tests (RATs) or polymerase chain reaction (PCR) laboratory tests. For example, Germany faced challenges in terms of shortages of PCR machines, in available labor to use the machines, and PCR testing materials since the beginning of 2022. However, these constraints were not solvable within the short-term. Therefore, the German Federal Ministry of Health announced its prioritization of PCR tests that are reimbursed by public funds. Specifically, it decided to prioritize symptomatic individuals [1]. In contrast, a positive RAT in asymptomatic patients was not recommended for follow-up with a PCR test when the incidence was high (> 1%) [1]. Moreover, to end isolation, a negative RAT was generally considered as sufficient [1]. Conversely, some experts argued that isolation should only be terminated using PCR testing because RATs are less accurate [2]. Moreover, some experts reasoned that symptomatic contacts of COVID-19 patients who had a positive RAT test result could be spared further testing [3].

Given the controversies around the prioritization of PCR tests, this study developed a mathematical model that defined priority groups for lab-based PCR testing. The model was applied in a German setting.

## Methods

To find a solution to the aforementioned prioritization problem, this study constructed a mathematical model. This model considers situations of limited PCR testing capacity but with an unlimited availability of RATs. The model assumes that PCR lab tests are the gold standard. Nevertheless, false negative test results could arise, for example, because the sample was taken too early or too late [4]. In addition, false positive test results have been reported. The PCR test value is thus determined as follows:

$$V\left(p\right)=-\left(1-{SP}_{PCR}\right)∙V_{TN}+p∙\left(1-p\right)∙{SE}_{PCR}∙V_{TP},$$ (1)

where *SP* denotes specificity, *V*_TN_ is the value of a true-negative diagnosis, *p* denotes the pre-test (i.e., a priori) probability of COVID-19, *SE* refers to sensitivity, and *V*_TP_ is the value of detecting (and isolating) a true-positive case. The first term defines the value of avoiding the costs of not diagnosing a true-negative case correctly. Costs are caused by an unnecessary isolation. The second term defines the positive value of diagnosing a true-positive case correctly. The value of identifying a true-positive case lies in preventing the transmission of COVID-19. The pre-test probability of a true-positive case is multiplied by its complimentary probability because the complimentary probability signifies the value created by PCR testing in a true-positive case (i.e., the value that goes above the pre-test probability). Hence, PCR testing yields a higher value when identifying a true-positive case with a low pre-test probability.

Given that RATs are an alternative to PCR testing, the model calculates the incremental value of PCR tests over RATs:

$$\begin{array}{c} V\left(p\right)=-\left(1-{SP}_{PCR}\right)∙V_{TN}+p∙\left(1-p\right)∙{SE}_{PCR}∙V_{TP}\\ -\left(-\left(1-{SP}_{RAT}\right)∙V_{TN}+p∙\left(1-p\right)∙{SE}_{RAT}∙V_{TP}\right), \end{array}$$ (2)

where *SP*_PCR_ > *SP*_RAT_ and *SE*_PCR_ > *SE*_RAT_. I considered estimates of sensitivity and specificity to be independent, because estimates of specificity even for RATs are typically very high. That is, the correlation between sensitivity and specificity was considered negligible. Eq 2 was differentiated with respect to *p* to determine the pre-test probability, which yields an optimal incremental value:

$$V^{'}\left(p\right)=\left(1-2p\right)∙{SE}_{PCR}∙V_{TP}-\left(1-2p\right)∙{SE}_{RAT}∙V_{TP}=0$$ (3)

⇔1-2p=0 (4)

$$\Leftrightarrow p^{*}=0.5.$$ (5)

**Fig 1:**
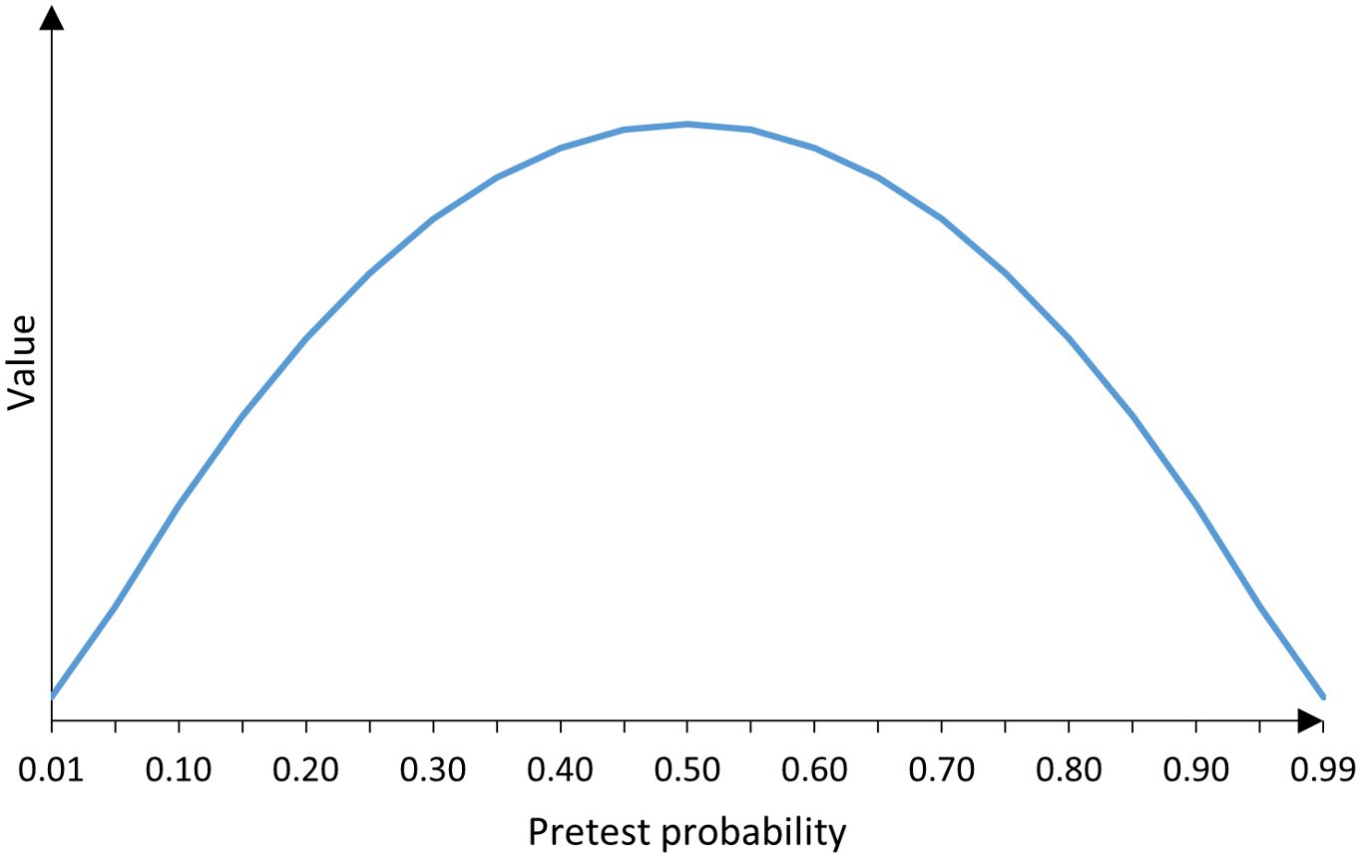
Relationship between the value of polymerase chain reaction testing and the pre-test probability.

Given that the second derivate of *V*(*p*) is negative, the value of PCR testing is maximized at a pre-test probability of 0.5. Based on the negative quadratic terms of Eq 2, the value function with respect to the pre-test probability is bell-shaped (see Fig 1). To determine lower and upper bounds for the pre-test probability, I analyzed two cases in which the lab-based PCR test-positivity rate in the population *μ* is either smaller or larger, respectively, than the value-maximizing probability p*.

Expanding the number of available PCR tests would allow testing individuals with a lower a priori probability, which reduces the test-positivity rate in the population *μ*. However, the expansion is constrained by the number of available PCR tests. The objective is thus to maximize the value of testing given the current test-positivity rate. The optimal pre-test probability interval includes the value-maximizing probability p* because testing at p* is most efficient. In general, the interval includes all individuals in whom testing value is larger than at the test-positivity rate.

Assuming the case where *μ* < p* yields the following lower and upper bounds for the threshold pre-test probability (*λ*_L_ and *λ*_U_, respectively):

$$\lambda_{L}=\left\{\begin{array}{cc} \mu +x\left(\mu -p^{*}\right), & \mu \geq -x\left(\mu -p^{*}\right)\\ 0, & \mu <-x\left(\mu -p^{*}\right) \end{array}\right.$$ (6)

$$\lambda_{U}=p^{*}+p^{*}-\mu ={2p}^{*}-\mu ,$$ (7)

where *x*]0, ∞) is a scaling factor that defines the share of the pre-test probability range below *μ*. If *x* = 1, one third of the pre-test probability range is below *μ* (if not bounded by zero) and covers half of the tested population. If *x* = 2, there is an equal share of the range above and below *μ*. The latter is justified by a lack of information about the distribution of a priori probabilities.

Based on Eqs 6 and 7 it follows that decreasing the availability of PCR tests or increasing the demand for testing (both resulting in a higher test-positivity rate) leads to an increase in the lower bound and a decrease in the upper bound of the pre-test probability.

Additionally, assuming the case where *μ* > p*, the following lower and upper bounds for the threshold pre-test probability are obtained:

$$\lambda_{L}={1-\mu }^{}$$ (8)

$$\lambda_{U}=\left\{\begin{array}{cc} \mu +x\left(\mu -p^{*}\right), & \mu +x\left(\mu -p^{*}\right)\leq 1\;\\ 1, & \mu +x\left(\mu -p^{*}\right)>1. \end{array}\right.$$ (9)

If *x* = 1, one third of the pre-test probability range is above *μ* (if not bounded by one) and covers half of the tested population. If *x* = 2, there is an equal share of the pre-test probability range above and below *μ*.

If *λ*_U_ − *λ*_L_ < *μ* − (*μ* − *λ*_L_), the range for the a priori probability is narrower than that for a uniform distribution of a priori probabilities. If the intention is not to strict access and if the same distribution as in Eqs 6 to 9 is assumed, the share of the population from which positive cases are drawn is set equal to *μ* − (*μ* − *λ*_L_). To satisfy *λ*_U_ − *λ*_L_ < *μ* − (*μ* − *λ*_L_), the updated upper threshold probability $\lambda_{U}^{u}$ was calculated as follows:

$$\lambda_{U}^{u}=\left\{\begin{array}{cc} \mu +\frac{\mu -\;(\mu -p^{*})}{2}, & \mu +\frac{\mu -\;(\mu -p^{*})}{2}\leq 1\\ 1, & \mu +\frac{\mu -\;(\mu -p^{*})}{2}>1. \end{array}\right.$$ (10)

The updated lower threshold probability $\lambda_{L}^{u}$ becomes:

$$\lambda_{L}^{u}=\left\{\begin{array}{cc} \mu -y\left(\frac{\mu }{2}-\frac{p^{*}}{4}\right), & \mu -y\left(\frac{\mu }{2}-\frac{p^{*}}{4}\right)\geq 0\\ 0, & \;\mu -y\left(\frac{\mu }{2}-\frac{p^{*}}{4}\right)<0, \end{array}\right.$$ (11)

where *y*]0,1] is a scaling factor that defines the size of the testing population with a pre-test probability below *μ* relative to *x*. If *y* = 1, the portion of the tested population below *μ* is the same as in Eq 6. That is, expanding the range of the pre-test probability does not lead to a more skewed distribution. If the distribution becomes more skewed, then *y* < 1 holds and the share of the population from which positive cases are drawn is still smaller than *μ* − (*μ* − *λ*_L_).

**Fig 2:**
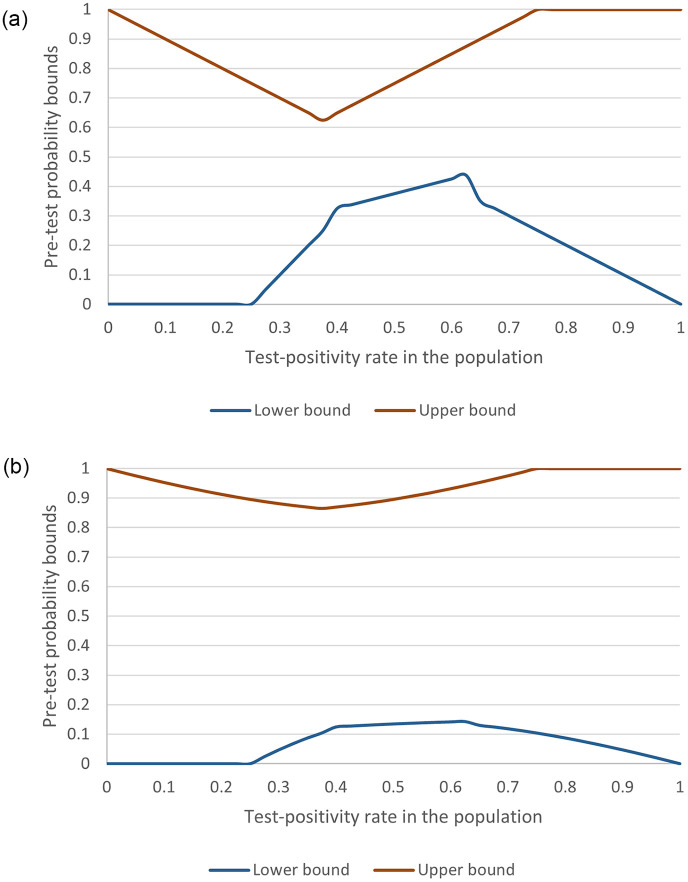
Lower and upper bounds for the threshold pre-test probability depending on the polymerase chain reaction test-positivity rate. (A) Contacts at normal risk. (B) Contacts at +100% risk.

Fig 2 shows the lower and upper bounds for the threshold pre-test probability depending on the PCR test-positivity rate. It is assumed that two thirds of the tested population have a pre-test probability below *μ*.

Given that *V*_TP_ depends on the individual’s number of contacts, their age, and various other risk factors, we are able to derive equivalent threshold probabilities for a higher number of contacts or contacts at higher risk for complications. If *V*_TP_ increases, the lower threshold probability decreases because PCR testing is then more valuable, even with a lower pre-test probability. The adjusted lower threshold probability $\lambda_{L}^{uu}$ was calculated as follows:

$$V\left(\lambda_{L},V_{TP}\right)=\lambda_{L}^{u}∙\left(1-\lambda_{L}^{u}\right)∙V_{TP}=\lambda_{L}^{uu}∙\left(1-\lambda_{L}^{uu}\right)∙V_{TP}^{high},$$ (12)

where $V_{TP}^{high}$ denotes the value of diagnosing a true-positive case with a higher number of contacts or contacts at higher risk for complications. Rearranging Eq 12 yields the following:

$${-(\lambda }_{L}^{uu}{)}^{2}+\lambda_{L}^{uu}-\lambda_{L}^{u}∙\left(1-\lambda_{L}^{u}\right)∙\frac{V_{TP}}{V_{TP}^{high}}=0.$$ (13)

Solving for $\lambda_{L}^{uu}$ yields:

$$\lambda_{L}^{uu}=\frac{-1\pm \sqrt{1-4{∙\lambda }_{L}^{u}∙\left(1-\lambda_{L}^{u}\right)∙\frac{V_{TP}}{V_{TP}^{high}}}}{-2}.$$ (14)

Following the reasoning for the lower threshold probability, if *V*_TP_ increases, the higher threshold probability increases because PCR testing is then more valuable even at a higher pre-test probability. Thus, the adjusted higher threshold probability $\lambda_{H}^{uu}$ is calculated as follows:

$$\lambda_{H}^{uu}=\frac{-1\pm \sqrt{1-4{∙\lambda }_{H}^{u}∙\left(1-\lambda_{H}^{u}\right)∙\frac{V_{TP}}{V_{TP}^{high}}}}{-2}.$$ (15)

Fig 2B shows the lower and upper bounds for the threshold pre-test probability depending on the PCR test-positivity rate and assuming contacts at 100% risk.

## Results

During the fifth calendar week of 2022, a total of 450,588 PCR tests for the diagnosis of COVID-19 were conducted daily in Germany [5]. The daily COVID-19 incidence was 133,173 on February 6, 2022 [6]. Therefore, the test-positivity rate was 30%. Based on Eqs 6 and 7 and assuming that at least two thirds of the tested population have a pre-test probability below the test-positivity rate, I obtained *λ*_L_ ≥ 0.1 and *λ*_U_ = 0.7. Hence, the minimum a priori probability of PCR testing in the general population was 10%. Next, I determined priority groups based on symptoms, exposure to COVID-19, and prior RAT results (see Table 1). I defined symptomatic people as having a 25% likelihood of testing positive. Even without a prior RAT, they would be eligible for PCR testing. However, if they test negative on an RAT, their negative predictive value is larger than 90% [7]; hence, they would be excluded from PCR testing. Furthermore, given that symptomatic individuals with a positive RAT have a positive predictive value of above 90% [7], they would also be excluded from PCR testing and could thus receive a second RAT instead. With the increasing scarcity of PCR tests and a lower threshold probability of above 25%, symptomatic people should only be tested by using RATs.

**Table 1:** Priorities for polymerase chain reaction (PCR) testing based on a PCR test-positivity rate of 30%.

	Asymptomatic			Symptomatic	
	General population	Contacts at +100% risk	Contacts of confirmed cases	General population	Contacts at +100% risk
RAT positive	+	+	+	-	-
RAT negative	-	-	-	-	-
No RAT	-	-	-	+	+

RAT, rapid antigen test; + priority;—no priority

For asymptomatic individuals with no known exposure to someone with COVID-19, a 1% pre-test probability approximating the 7-day incidence on February 7, 2022, was assumed. In this population, PCR testing would be valuable only after a positive RAT result.

Health-care workers, those working in care homes with older patients, first responders (i.e., police officers, firefighters, military personnel, paramedics, medical evacuation pilots, dispatchers, nurses, doctors, emergency medical technicians, and emergency managers), and contacts of confirmed cases (especially family members) were assumed to have a 5–10% likelihood of testing positive [7]. Herein, PCR testing after a positive RAT result would only be valuable in individuals with a 5% likelihood of testing positive prior to RAT testing.

For individuals who have twice the number of contacts or who have contacts with twice the usual risk, the lower testing threshold is 4.7%. In other words, the lower testing threshold is reduced to less than half. Conversely, the upper testing threshold increases to 88%. However, the recommendations for PCR testing for the defined prior probabilities do not change (see Table 1). The only exception are contacts of confirmed cases with a 10% likelihood of testing positive as positive RAT results should then be confirmed using PCR testing.

## Discussion

To address lab-based PCR test bottlenecks resulting from high COVID-19 incidence rates, the model developed in this study maximizes the value of positive testing. Fundamentally, merely maximizing the number of positive tests in the testing population does not consider the pre-test probability of COVID-19 and hence the additional value of positive testing. Specifically, the model recommends using RATs to test asymptomatic individuals with no known exposure to COVID-19 and PCR testing in symptomatic people (in accordance with Du et al. [8]). Furthermore, positive RAT results from asymptomatic individuals with no known exposure to COVID-19 should be confirmed using a PCR test. Hence, using RATs among asymptomatic individuals with no known exposure to COVID-19 would be considered a triage tool that enriches the population taking a PCR test (cf. [9]). The notion of using PCR as a confirmatory test is corroborated with that of another COVID-19 testing model [10].

In contrast to previous models, however, this study defines the value-maximizing pre-test probability, shape of the value function, and upper and lower probability thresholds for priority PCR testing. Hence, individuals with pre-test probabilities outside the probability thresholds are not prioritized. This supports the idea that the “opportunity cost of a test exceeds the value of information for individuals with pre-test probabilities close to 0 or 1, and thus near certain types are left untested” [10].

The study by Smits et al. [11] made a valuable contribution by presenting a utility function for a diagnostic test. In contrast to my study, their research measured the value (utility) of obtaining outcomes such as a true-negative or true-positive diagnosis. However, their value (utility) function does not determine the incremental value of one diagnostic test compared to another.

As a word of caution, the analysis implicitly assumes that the value of a positive PCR test is the same both for asymptomatic and symptomatic individuals. The value of a positive PCR test lies in reducing the transmission rate by isolating infectious individuals [12]. While the U.S. Centers for Disease Control and Prevention [13] considered infectiousness of asymptomatic individuals to be 25% lower relative to symptomatic patients (in the pre-Omicron era), asymptomatic individuals may transmit more than symptomatic individuals if they are unaware of their infection [14]. It is not clear to what degree these factors cancel out, that is, yield a value of a positive PCR test that is different for asymptomatic and symptomatic individuals. To incorporate transmission probability in the model would require adjusting the pre-test probabilities. In any case, the fundamental notion that the value of PCR testing is maximized at a pre-test probability of 0.5 remains.

Changes in incidence lead to changes in the PCR test-positivity rate and, hence, influence the threshold for PCR testing. Therefore, the results of the application study present a snapshot and should not be extrapolated to different incidences. Nevertheless, for the same PCR test-positivity rate, results are transferable to different settings and countries.

This study assumed an unlimited availability of RATs. Future research may thus address a simultaneous RAT testing constraint.
